# How do memory systems detect and respond to novelty?

**DOI:** 10.1016/j.neulet.2018.01.053

**Published:** 2018-07-27

**Authors:** Alex Kafkas, Daniela Montaldi

**Affiliations:** Memory Research Unit, School of Biological Sciences, Division of Neuroscience & Experimental Psychology, University of Manchester, UK

**Keywords:** Novelty, Familiarity, Dopamine, Norepinephrine, Acetylcholine, Hippocampus

## Abstract

The efficiency of the memory system lies not only in its readiness to detect and retrieve old stimuli but also in its ability to detect and integrate novel information. In this review, we discuss recent evidence suggesting that the neural substrates sensitive to detecting familiarity and novelty are not entirely overlapping. Instead, these partially distinct familiarity and novelty signals are integrated to support recognition memory decisions. We propose here that the mediodorsal thalamus is critical for familiarity detection, and for combining novelty signals from the medial temporal lobe cortex with the relative familiarity outputs of computations performed in other cortical structures, especially the prefrontal cortex. Importantly, we argue that the anterior hippocampus has a prominent role in detecting novelty and in communicating this with midbrain and striatal structures. We argue that different types of novelty (absolute or contextual) engage different neurotransmitter systems that converge in the hippocampus. We suggest that contextual or unexpected novelty triggers dopaminergic hippocampal-midbrain coupling and noradrenergic-mediated pupil dilation. In contrast, absolute novelty triggers cholinergic-mediated hippocampal encoding accompanied by diminished pupil dilation. These two, distinct hippocampal encoding mechanisms both lead to later recollection but are sensitive to different types of novelty. We conclude that this neurotransmitter-mediated hippocampal encoding establishes the hippocampus in an encoding mode that briefly prevents the engagement of retrieval.

## Highlights

• Novelty is heterogeneous; heterogeneity driven by type of information that is novel.
• Familiarity and novelty signals originate from non-overlapping brain regions.
• These distinct signals combine to produce a relative familiarity signal.
• Anterior hippocampal novelty-detection triggers neurotransmitter-mediated encoding.
• Encoding contextual novelty is dopaminergic/noradrenergic while absolute novelty is cholinergic.

## 1 Introduction

Familiarity and recollection and to a lesser extent, novelty, have been studied extensively in relation to the function of the structures of the Medial Temporal Lobes (MTL) including the hippocampus, the perirhinal (PRC), the entorhinal (ERC) and parahippocampal (PHC) cortices and the amygdala. The hippocampus has a central role in memory within this network of interconnected structures as is clearly evident from the severe amnesia that occurs following hippocampal damage [1,2]. Recent findings indicate that the structures of the MTL, although having somewhat unique roles within this network, work cooperatively with one another [3,4], as well as with an extended brain network [5,6], to promote novelty detection, memory encoding and retrieval. In this review, we will present evidence for the role of the hippocampus (and the rest of the MTL) in novelty detection and the relationship between this and its role in memory processing. Before, we discuss the neural evidence, we will start by defining the diverse meaning of novelty and we will provide a theoretical model, based on empirical findings, regarding the way familiarity and novelty signals contribute independently to recognition decisions. Subsequently, the neural evidence will be presented and a special emphasis will be placed on the potential role of the anterior hippocampus as a novelty detector and its ability to communicate with other important structures within the novelty network. We will propose a dual mechanism for the way novel information is encoded and learnt, in a way that supports later recollection, taking into account dopaminergic, noradrenergic and cholinergic inputs to the hippocampus.

## 2 What is novelty detection, and what is it detecting?

Novelty detection results in a cascade of neural responses and behavioural outcomes that highlight its evolutionary significance [7] and enable exploration [8] and flexible memory encoding of the novel information [[9], [10], [11]]. However, this novelty response is soon lost as repeated exposure to novelty results in fast neural adaptation across the novelty network [12]. Novelty detection is therefore associated with a series of distinct, although interrelated, processes each playing a unique role; from the initial evaluation of a stimulus, the generation of mismatch signals, and where relevant, the monitoring of unexpected outcomes, the integration of novel stimuli into pre-existing representations and thus the creation of new representations [13,14]. Each of these processes is key to *novelty detection* but the specific function and neural substrate of each process, remains, to a great extent, unspecified.

Therefore, novelty describes an attribute we can apply to a stimulus (however complex), when it lacks a pre-existing representation. However, there are different sources, or types, of novelty that are differentiated and determined by the nature of pre-existing representations. Therefore, a stimulus may be novel because it has not been experienced before and in this case, the novelty is for the stimulus itself. We will call this absolute novelty to discriminate it from contextual novelty. Contextual novelty, is the detection of novelty arising from a mismatch between the components of an encountered stimulus-context pairing. Context can be defined in terms of spatio-temporal or other information, that when repeatedly paired with a stimulus, or stimulus type, creates a representation. This may influence the way the stimulus, or stimulus type, is later processed, whether alone, in that context or in another context. Therefore, in an experimental environment, contextual novelty may be triggered by stimuli that are incongruent with the properties of an established context, or with neighbouring or concurrently presented stimuli, which serve to establish a predictive context. In the present paper, we focus on absolute and contextual novelty as types of episodic novelty (i.e., related to episodic memory); consideration of conceptual or semantic novelty (e.g., novel semantic concepts) is beyond the scope of this paper.

The role of the hippocampus and adjacent PRC in novelty detection has been explored over many years [13,15]. However, novelty detection engages a network of brain regions (see Section 4 below) whose functional significance remains underexplored. Importantly, the degree to which the hippocampus, along with the other brain regions that contribute to novelty detection, might respond differently to different types of novelty has not been investigated systematically [but see, 16]. Moreover, models of recognition memory, and its neural bases, have generally overlooked the possibility that novelty may not simply be ‘no familiarity’ but instead, novelty and familiarity may be two somewhat independent functions that offer distinct contributions to recognition memory decisions. Before we examine the role of the hippocampus, and related structures, in novelty detection, we will discuss the relationship between familiarity and novelty detection and their contribution to recognition memory.

## 3 How might novelty and familiarity signals combine to contribute to recognition memory?

There is a consensus that recognition memory can be supported by two kinds of memory: familiarity and recollection [[17], [18], [19], [20]]. Familiarity is the feeling that a stimulus has been encountered before, without the retrieval of additional contextual details about the encounter, while recollection involves the cued recall of additional non-stimulus information associated with the cueing stimulus. Interestingly, although cognitive models of memory incorporating aspects of novelty have been proposed, to date, no theory of memory has explicitly focused on the contribution of novelty detection to the evaluation of recognition memory. Previous models, for example, stress the role of novelty in learning new information and in memory updating [e.g., [21], [22], [23]], but they do not consider how the novelty signals combine with familiarity signals to inform recognition decisions (or memory decisions in general). Importantly, as will be discussed below, unlike previous cognitive/memory models, here (and in the relevant empirical work,) we propose that familiarity memory depends on the dynamic interaction between novelty and familiarity brain signals.

**Fig. 1:**
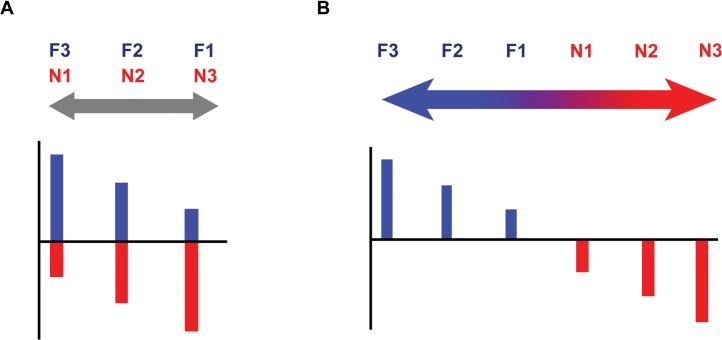
Relationship between familiarity and novelty according to the standard view. A) Familiarity and novelty as “mirror images” explain the behavioural output, i.e., something that is more familiar is less novel and vice versa. B) The familiarity – novelty continuum that describes the hypothetical neural response to familiarity and novelty if the two rely on the same neural substrates (consistent with the “mirror image” view). If the full continuum explanation is correct, assuming three levels to familiarity (F) and novelty (N) strength, a graded brain response should be expected honouring the whole scale: F3 (strong F) – F2 (moderate F) – F1 (weak F) – N1 (weak N) – N2 (moderate N) – N3 (strong N). Increased activity with increased familiarity strength is illustrated, but the opposite (i.e., increased activity with increased novelty strength) is also possible.

The standard use of the terms ‘familiarity’ and ‘novelty’ implies that something that is highly familiarity is by definition not novel and vice versa. We have argued [5,24] that a simple ‘mirror-image’ relationship between familiarity and novelty only explains the final, behavioural output of a recognition decision (i.e., something judged as familiar is not judged as novel; Fig. 1A), and does not accommodate findings describing familiarity and novelty signals in the brain. If the brain treats familiarity and novelty as mirror images then there have to be brain regions that respond to *both* familiar and novel stimuli in a graded manner, either responding maximally to the most familiar or to the most novel stimulus, but honouring a full continuum; from strong familiarity to strong novelty (Fig. 1B). Instead, drawing on fMRI and pupillometry data, we have reasoned [5,24] that the evidence so far is more consistent with a dual mechanism supporting the *interaction* between separate familiarity and novelty signals.

**Fig. 2:**
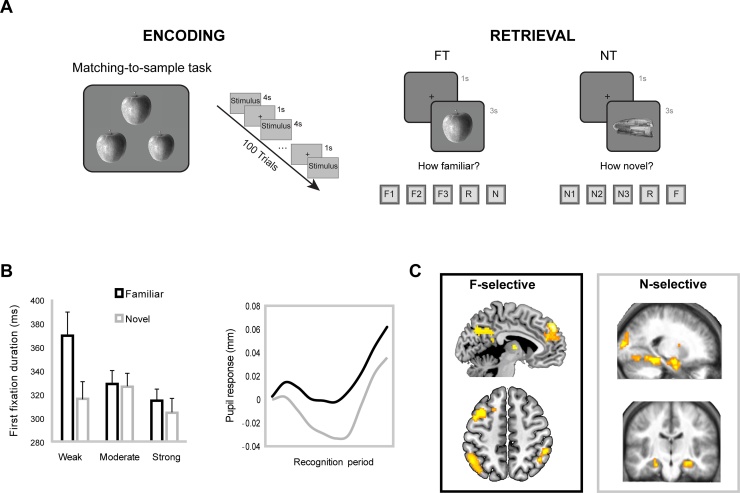
Exploring familiarity and novelty detection: Eye tracking and brain responses. A) Participants encoded single objects using a perceptual matching-to-sample task. At retrieval, inside the MRI scanner [5] or while undergoing eye tracking recording [24], participants engaged in two alternating tasks emphasising either familiarity detection (FT) or novelty detection (NT). A rating scale was provided in both tasks to evaluate strength of familiarity or novelty and to indicate instances of spontaneous recollection and correctly rejected stimuli (new items in FT; old items in NT). B) The duration of the first fixation and the pupil response discriminated between familiar and novel stimuli. C) Familiarity-selective and novelty-selective activation patterns were identified in non-overlapping brain regions. Familiarity-selective regions included the mediodorsal thalamus, the dorsolateral and superior medial prefrontal cortex, the anterior cingulate and the left angular gyrus. Regions along the ventral visual stream and critically the anterior hippocampus belong to the novelty-specific network. F1 = weak; F2 = moderate; F3 = strong familiarity; N1 = weak; N2 = moderate; N3 = strong novelty. Figure adapted from [5,24].

The idea that the neural substrates sensitive to detecting familiarity and novelty are not entirely overlapping is supported by findings from single neuron activity studies with experimental animals [[25], [26], [27]] and humans [28,29]. Consistent with the single neuron findings, evidence from fMRI shows that familiarity and novelty activation effects are identified in non-overlapping brain regions [30,31]. To directly explore this, recent work [5,24] employed a paradigm in which participants were asked to rate familiarity and novelty of old and new stimuli under two conditions; emphasising either familiarity or novelty detection (Fig. 2A). Subjective feelings of familiarity and novelty were rated on a scale from 1 to 3, with 1 being weak, and 3 being strong familiarity or novelty, respectively for each condition. Recollection responses were reported using a separate response and excluded from the targeted familiarity − novelty analyses.

The pattern of brain activity revealed the existence of separate familiarity- and novelty- sensitive brain regions (Fig. 2C) that, nevertheless, appear to interact and converge at key brain sites. Specifically, three classes of brain responses were identified: a) regions selectively sensitive to either familiarity or novelty as revealed by monotonic increases or decreases in activity with reported familiarity or novelty strength, b) regions showing a *relative* familiarity effect, sensitive to the full familiarity-novelty continuum from very strong novelty to very strong familiarity and c) regions sensitive to reported strength irrespective of the status of the stimulus as old or new. Importantly, these effects were not driven by any differences in performance levels as performance on familiarity and novelty decisions was matched. These findings strongly suggest that familiarity and novelty signals are, at least in part, non-overlapping. Consistent with these findings and the proposal of Kafkas and Montaldi [5] that familiarity and novelty signals make independent contributions to familiarity-based recognition, a more recent study [32] also showed that separate cortical and subcortical sources of familiarity and novelty activity contribute independently to recognition memory performance.

It is worth noting that the processing of familiar and novel stimuli also triggers distinct pupillary response patterns (Fig. 2B) [24,33] perhaps driven by the differential engagement of the brain’s familiarity-specific and novelty-specific networks. Moreover, fixation patterns, and especially the duration of the first fixation, clearly discriminates between weakly familiar and weakly novel stimuli within 320 ms of stimulus onset, with no significant differences in reaction time (Fig. 2B) [24]. This point, between weakly familiar and weakly novel responses, is the point on the continuum where the intersection between novelty and familiarity occurs; where the behavioural responses, while accurate, are less confident. Thus, these robust findings further support the suggestion that separate mechanisms may well underpin novelty and familiarity detection.

**Fig. 3:**
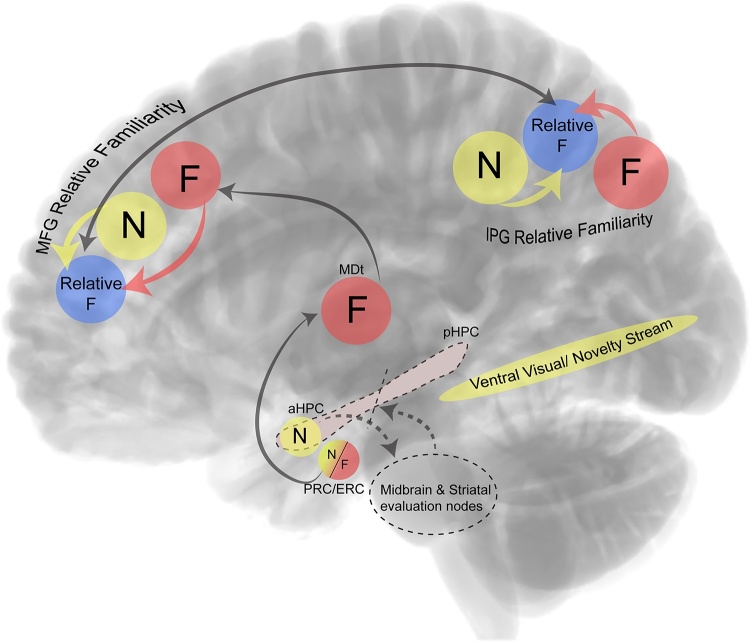
Integration of familiarity and novelty signals in the brain. Computations from novelty-selective and familiarity-selective regions converge to provide a *relative* familiarity output. The mediodorsal thalamus (MDt) plays a central role in detecting familiarity and in orchestrating convergence of novelty from the medial temporal cortical areas to middle prefrontal regions (Middle Frontal Gyrus, MFG). MFG interacts with the inferior parietal gyrus (IPG), where familiarity- and novelty-sensitive regions also converge to provide a *relative* familiarity output. The anterior hippocampus detects novelty and conveys novelty-related information to midbrain and striatal structures for salience evaluation.

Familiarity and novelty signals, therefore, appear to be generated in non-overlapping brain regions, drawing on separate mechanisms. Critically, the familiarity and novelty signals converge to provide a *relative* familiarity output (Fig. 3). The evidence for this proposal is further discussed in the next section.

## 4 Two distinct familiarity and novelty networks

Regions lying along the ventral visual stream have generally been considered to belong to the novelty network. In particular, in the study discussed above [5] the novelty network (i.e., regions that responded selectively to increased levels of reported novelty) included the primary and secondary occipital cortex, the fusiform gyrus and critically the PRC and anterior hippocampus (Figs. 2C and 3). Similar regions have been identified in other studies exploring novelty detection using different experimental paradigms [e.g., [34], [35], [36], [37]]. Indeed, different types of novelty (stimulus absolute novelty, conceptual novelty, context novelty) have been shown [38] to activate similar, and to a great extent overlapping, brain regions, most prominently across the ventral visual stream, as in [5], despite key paradigm differences. Interestingly, fMRI shows that the engagement of regions in novelty detection tasks, and especially the medial PFC, the anterior hippocampus, and regions along the ventral visual stream, decline with age [39,40]. Therefore, dysfunction of brain regions that appear critical for novelty processing, and underlie successful memory encoding of novel information, may go some way to explaining age-related memory decline. However, the specific functional role of the brain regions responding to novelty is not well-specified. Further research may help characterise the specific novelty-related functions subserved by the regions within the novelty network, although one should expect that their contribution would not be limited to novelty detection.

Familiarity selective effects were identified in the mediodorsal thalamus, the dorsolateral and superior medial prefrontal cortex, the anterior cingulate and the left angular gyrus (Fig. 2C). It is critical to note that these two networks are selective to either novel or familiar stimuli, while convergence of the two was observed in lateral and medial frontal, and inferior parietal regions, which were shown to be sensitive to *relative* familiarity (i.e., across the whole continuum) of both novel and familiar stimuli (Fig. 3).

### 4.1 The thalamus as an integrator of familiarity and novelty signals

The thalamus, and especially the mediodorsal thalamic nucleus (MDt), is part of the extended MTL cortical network that supports familiarity memory [41]. We have shown [5], that the MDt has a prominent role in detecting increasing levels of familiarity (Figs. 2C and 3) and this is consistent with other neuroimaging and neuropsychological evidence that emphasises the role of this thalamic nucleus in familiarity-based recognition [33,42, for a review see, 43]. Importantly, recent evidence suggests that the MDt region appears to have a material-independent role, showing functional connectivity across material-specific regions within the PRC and PHC during the processing of familiarity decisions [44]. This critical role of the MDt in computing familiarity signals is consistent with its extensive connectivity with dorsolateral, ventrolateral and medial PFC regions [45, for a review see, 46], identified by Kafkas and Montaldi [5] as supporting relative familiarity judgments. Based on this evidence, we propose here that the MDt acts as a critical hub of information integration for the processing of familiarity-based recognition. This proposal is consistent with the accumulating evidence for the *active* role for the thalamus in information integration and the promotion of subcortical-cortical and cortico-cortical communication [e.g., [47], [48], [49], [50], [51]]. According to our proposal here, and as shown in Fig. 3, the MDt orchestrates familiarity decisions by combining information from novelty-sensitive processing in the MTL cortex (PRC and PHC) and the relative familiarity computations performed in the PFC. The lateral and medial inferior parietal regions show similar activation patterns to the PFC and are likely, therefore, to support a similar role in computing the relative familiarity of both familiar and novel stimuli [5]. However, the relatively limited connections between the MDt and parietal regions [52] suggest that the parietal relative familiarity role is likely mediated indirectly via its extensive connectivity with the PFC [e.g., 53] (Fig. 3) (although additional connectivity with the MTL may also contribute).

### 4.2 PRC, anterior hippocampus and midbrain structures: novelty detection and evaluation

A contribution of the anterior hippocampus to novelty detection has been reported in numerous studies [e.g., 31,32,37,[54], [55], [56], [57]] but its functional significance remains unclear. The anterior hippocampus appears to be tuned to discriminate between novelty and familiarity responses even at low levels of confidence as significantly greater activation accompanies weak novelty than weak familiarity [unpublished observation from the data in 5]. This pattern suggests the rapid engagement of a familiarity and novelty discrimination system in which the anterior hippocampus plays a critical role in detecting novelty, even when it is weak.

The majority of studies exploring novelty, report that novelty detection engages both the anterior hippocampus and the anterior parahippocampal gyrus, especially the PRC. This consistent pattern suggests that both regions form part of the novelty network, which may appear to be at odds with the dominant view that the PRC and the hippocampus contribute very differently to recognition memory decisions [20,41]. However, as noted earlier, novelty displays itself in many ways and it is therefore likely that its detection draws on several processes (as does the detection of familiarity). Moreover, the PRC and the anterior hippocampus exhibit similar connectivity profiles [58] and are therefore very well placed to work together, while potentially making distinct contributions to memory in response to novelty detection. We therefore propose here that while the PRC and anterior hippocampus are both sensitive to novelty signals, these signals are used to drive somewhat independent mechanisms. The novelty signals computed in PRC (and parahippocampal cortex) contribute selectively to a familiarity memory mechanism, and through the above-mentioned MDt-PFC network, responsible (with input from parietal cortex) for combining novelty and familiarity signals, generate an estimate of the *relative* familiarity of stimuli. This is consistent with evidence from animal [e.g., 26,59,60] and neuroimaging studies [e.g., 33,61] regarding the role of the PRC in familiarity memory. Indeed, in a recent study [4] material-selective familiarity effects were isolated in the PRC and ERC, for objects and the PHC for both objects and scenes. Also, the PRC is active when strong familiarity is reported, and even when compared to equally strong recollection [33]. Finally, lesions in rat PRC impair discrimination between familiar and novel stimuli, while novelty detection (i.e., exploration of pairs of novel items) remains intact [60,62]. Taken together, these findings converge to suggest that the PRC and hippocampal signals differ in that the PRC novelty signal supports the detection and evaluation of relative familiarity memory, while, as will be discussed in detail below, the anterior hippocampal novelty signal triggers the exploration and encoding of novel information.

The role of the novelty signal generated in the anterior hippocampus, we therefore argue, is distinct from that generated in the PRC, as it relates directly to the role of the hippocampus (anterior and posterior) in constructing new relational memories. In particular, the anterior hippocampus contributes to the integration of new information with that of already formed associations between events [63,64]. We propose that the specific role played by the anterior hippocampus is underpinned by its close functional connectivity with midbrain and striatal regions [65,66], whereby the anterior hippocampus, and especially CA1, which is particularly densely represented in the anterior part of the hippocampus [67], communicates the novelty signal to the midbrain and the striatum (Fig. 3) [68]. Therefore, the anterior hippocampus holds a ‘memory updating’ role by conveying critical novelty-related information to midbrain and striatal structures, which mediate the evaluation of the signal.

Indeed, memory updating has previously been linked to enhanced functional connectivity between the MTL and the midbrain [68]. Although, this network of hippocampal-striatal/midbrain connections has been systematically studied in relation to reward anticipation and reward learning [e.g., [69], [70], [71]], other studies have demonstrated that explicit reward or feedback manipulation is not a prerequisite for the engagement of the hippocampal-striatal/midbrain regions during learning or memory updating [68,72,73]. Therefore, the functional coupling between the dopaminergic midbrain and the MTL (most prominently the hippocampus) is not a function of the rewarding value of the stimuli, although reward may result in engagement of this network. Instead, we argue that the midbrain-hippocampal network is particularly tuned to detecting, evaluating and encoding contextual novelty. Indeed, we demonstrated [68] that although the anterior hippocampus was active for absolute novelty [see also, 5], it was the contextual novelty of the stimuli that drove the functional connectivity between the midbrain and the hippocampus. Moreover, in the same study, robust substantia nigra/ventral tegmental area (SN/VTA) activation was found when unexpected familiarity (i.e., contextual novelty) was contrasted with expected absolute novelty. This strongly suggests that the novelty-related dopaminergic modulation of the hippocampus is a function of the expected, or surprising, nature of the stimuli within the current context.

Therefore, the novelty signal in the anterior hippocampus [5,74] is communicated to SN/VTA for novel stimuli of particular salience; for example, in the case of contextual novelty, unexpected or surprising information, or a particularly rewarding stimulus. The factor common to all these types of stimuli is that they are motivational, potentially guiding future action, and therefore the integration of the presented information with existing knowledge is pivotal. This integration is achieved through the bidirectional connections that characterise the hippocampal-midbrain circuit. According to the influential model proposed by Lisman and Grace [65] dopamine release from the midbrain and specifically the SN/VTA converges on the hippocampus, triggering long-term potentiation and enabling new learning. As highlighted above, this mechanism appears to be specialised for motivationally significant stimuli, and in the case of novelty detection, is implicated when unexpected, (i.e., contextually novel) information is detected. The ensuing memory encoding is likely to involve both the anterior and the posterior hippocampus since the enhanced SN/VTA- hippocampal connectivity involves both anterior and posterior aspects [68]. This is consistent with recent evidence supporting the contribution of anterior and posterior hippocampus to encoding [75], and provides an explanation for previously reported inconsistences regarding the selectivity of the role of the anterior hippocampus in novelty detection/encoding [30,74,[76], [77], [78], [79]]. Therefore, the initial detection of novelty (either absolute or contextual) engages only the anterior hippocampus, but then both anterior and posterior hippocampal encoding of novel information occurs following midbrain/striatal dopaminergic facilitation.

### 4.3 Distinct hippocampally-mediated novelty-driven encoding mechanisms: dopaminergic, noradrenergic and cholinergic contributions

**Fig. 4:**
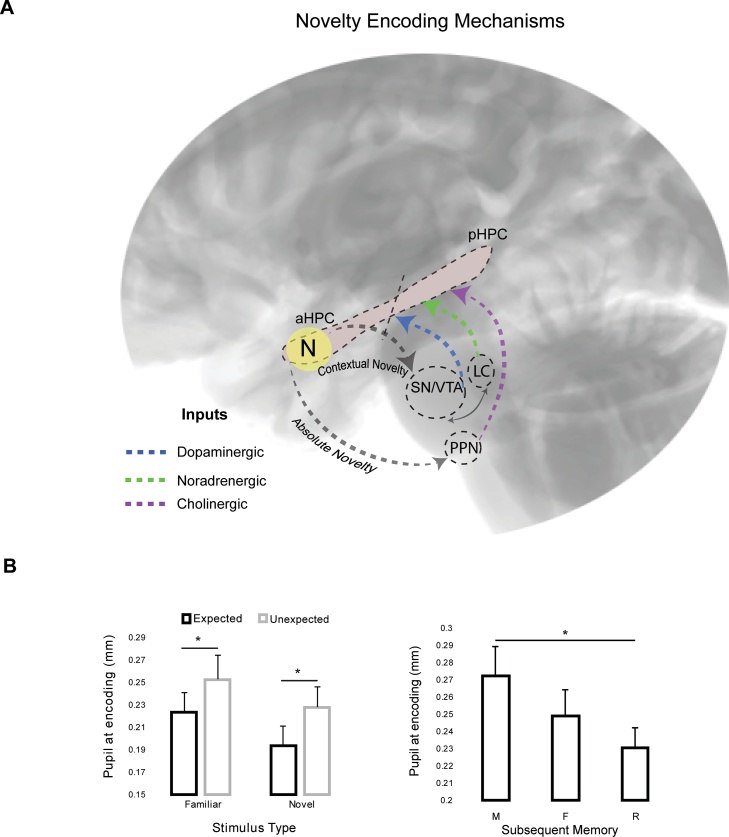
Anterior hippocampal novelty detection and associated neurotransmitter-mediated encoding mechanisms for absolute and contextual novelty. A) Detection of contextual novelty engages the dopaminergic SN/VTA [65,68] and affects the sympathetically innervated pupillary response via increased engagement of the noradrenergic system and especially the locus coeruleus (LC). This results in dopamine and norepinephrine release in the hippocampus and ensures effective learning and integration of new information with pre-existing knowledge. Detection of absolute novelty [80] engages the anterior hippocampus but in this case the cholinergic parasympathetic system facilitates learning. The novelty signal triggers the release of acetylcholine into the hippocampus from the pendunculopontine nucleus (PPN) of the midbrain, possibly via the basal forebrain (not shown in the Figure). Both encoding mechanisms enable efficient associative encoding resulting subsequently in recollection of information but are triggered by different types of novelty detected in the anterior hippocampus. B) Contextual and absolute novelty at encoding are accompanied by distinct pupil response patterns although in both cases later memory is supported by recollection. Contextual novelty results in increased phasic pupil dilation [left panel; 68], while absolute novelty is accompanied by a tonic pupil response, characterised by diminished dilation for subsequently recollected stimuli [right panel; 80]. *p < .05. Panel B of the Figure adapted from [68,80].

According to the explanation presented above the role of the anterior hippocampus is to provide efficient, graded, novelty detection, and to communicate the significance of the novelty to the striatum and the dopaminergic midbrain, where its salience is further evaluated. The hippocampus is subsequently re-engaged via the hippocampal-midbrain circuit, and dopaminergic release triggers LTP affecting both anterior and posterior hippocampal regions (Fig. 4). This enables effective learning of the contextually novel information and perhaps integration of episodic learning with pre-existing knowledge and/or experience.

As described above the hippocampal-midbrain circuit is selectively engaged in the case of contextual novelty but not in the case of stimulus absolute novelty [68]. Nevertheless, absolute novelty also activates the anterior hippocampus [e.g., 5,37,76], but in this case it does so directly and no dopaminergic involvement is required. This means that there are potentially two encoding mechanisms that engage the hippocampus and lead to the subsequent recollection of previously novel information. The key difference is that the first mechanism, as described above, prioritises contextual novelty and is dopaminergic, while the second is sensitive to novel information without the need for motivational significance (Fig. 4A).

Indirect evidence for the existence of two distinct encoding mechanisms comes from recent eye-tracking and pupillometric data (Fig. 4B). We have shown [68] that the detection of contextual novelty, characterised by increased hippocampal-striatal/midbrain connectivity, was accompanied by increased visual exploration (increased number of fixations) and increased pupil dilation (Fig. 4B). A follow-up recognition task revealed that the later recognition of these contextually novel stimuli was characterised by increased levels of reported recollection (compared to contextually expected stimuli). Therefore, increased pupil dilation is associated with the dopaminergic control of memory formation, driven by the hippocampal-midbrain circuit. On the other hand, we also showed that the encoding of novel stimuli (i.e., information with absolute novelty), that are not unexpected or contextually novel, but are also later recollected, are accompanied at encoding by *reduced* levels of pupil dilation (or pupil constriction) relative to those stimuli which are subsequently found to be weakly familiar or forgotten (Fig. 4B) [80, for a similar effect see 81]. This is highly consistent with the well-documented reduced pupil dilation levels characterising absolute novelty relative to familiarity (the pupil old/new effect) [24,33,82].

The pupillometric effects accompanying the encoding of contextual novelty (increased pupil dilation) and those accompanying the encoding of absolute novelty (decreased dilation or pupil constriction) may therefore, be controlled by different neural systems. Indeed, we propose here that these contrasting pupil effects characterising different forms of novelty-related encoding, which both support later recollection, are controlled by the engagement of different neurotransmitter systems (Fig. 4A). In the case of contextual novelty, dopaminergic release in the midbrain and striatum affects the sympathetically innervated pupillary response via increased engagement of the noradrenergic system and the locus coeruleus (LC), where the majority of noradrenergic neurons are located in the brain [83]. Norepinephrine release from LC has been linked to increased phasic pupil dilation [[84], [85], [86]], the attentional prioritisation of significant stimuli [[87], [88], [89]] and more recently to novelty detection [90]. Furthermore, norepinephrine production relies on the conversion of dopamine [91] and the two systems are seen as working in a complementary fashion to support learning and decision making [92,93]. Although there will be functional differences between the contributions of the noradrenergic system and the dopaminergic system to motivational learning [94], they both appear to support cognitive processes that are engaged in the detection of contextual novelty and the triggering of related encoding; including, motivation, evaluation, reward and prediction (dopaminergic) [[95], [96], [97], [98], [99]], and attentional shift, cognitive flexibility, effortful engagement and response to challenge or arousal (noradrenergic) [[100], [101], [102], [103]]. Critically, the hippocampus is a target of both neurotransmitter pathways; underpinning its functional connectivity with midbrain and LC structures [83,104,105]. Therefore, contextual novelty triggers dopaminergic hippocampal-midbrain strengthening and noradrenergic-mediated phasic pupil dilation, which also involves LC-hippocampal interactions.

In contrast, the encoding of absolute novelty does not draw on either the dopaminergic or the noradrenergic systems. Instead, here, encoding that leads to recollection is accompanied by a tonic pupil response, characterised by reduced dilation [80], potentially due to disengagement or inhibition from the LC-noradrenergic system [83,84]. The pupil constriction accompanying this encoding [80,81] (Fig. 4B), is controlled by the parasympathetic nervous system [106], which is predominantly driven by cholinergic neurotransmitter pathways (Fig. 4A). Indeed, there is a well-established link between acetylcholine function and learning and memory [107,108], and disruption of cholinergic afferents disrupt hippocampal-mediated learning [109]. Moreover, acetylcholine increases in hippocampal CA1 when novelty is detected [110]. Therefore, we believe that this cholinergic hippocampal novelty detection and consequent encoding is selective to absolute (as opposed to contextual) novelty and results in the parasympathetic (cholinergic) control of pupil response. We have proposed before [80] that this reduced pupil dilation (or increased pupil constriction) characterising effective memory formation when absolute novelty is detected (and encoded), reflects the restriction of internal processing to the encoding of the novel information. Interestingly, a similar function has been proposed for the role of acetylcholine in learning by Easton et al., [111], relating it to the encoding prioritisation of novel information in the hippocampus. According to this model the role of acetylcholine-mediated hippocampal novelty response and encoding is to restrict the attentional focus and to aid the active suppression of retrieval of interfering information during learning. Therefore, the second encoding mechanism we propose is engaged in when absolute novelty is detected, and triggers acetylcholine-mediated hippocampal encoding (accompanied by diminished pupil dilation/constriction) that leads to later recollection (Fig. 4).

In summary, we propose that novelty detection engages two distinct encoding mechanisms involving the hippocampus. We stress the distinction between contextual novelty and absolute novelty in terms of the different neurotransmitter-enabling mechanisms that support them, whose triggered encoding leads to similar recollection outcomes (although exactly what information is recollected and what further memory systems might be activated remains to be explored). Further fMRI and combined pharmacological-fMRI evidence will be needed to explore the conditions under which dopaminergic/noradrenergic and cholinergic inputs to the hippocampus promote learning.

As we have established in this section, novelty detection critically engages the hippocampus ensuring the effective learning of new information. But, it also supports retrieval, and especially recollection. The dual role of the hippocampus in both novelty detection/encoding and in retrieval has challenged researchers for decades, and many theories regarding the potential specialisation of hippocampal regions, or subfields, have been put forward [for recent proposals and discussions see 78,112]. While not a focus of this particular review, there may be value in linking this question to our proposed specialisation of neurotransmitter pathway-driven hippocampal encoding. It is likely that novelty detection triggers an encoding mode in the hippocampus via dopamine, norepinephrine or acetylcholine release, depending on the type of novelty detected. In contrast, when no such release is triggered, the hippocampus may return to a retrieval mode. According to this argument, hippocampal structures would be critical for both encoding and retrieval, but their mode of function (i.e., their performed computations) would differ. Some recent evidence supports this proposal [75,113].

## 5 Conclusions and summary

We draw on our previous argument that familiarity and novelty signals in the brain originate from somewhat distinct sources, and propose that the way familiarity and novelty signals can be both distinct and integrated is critical to our understanding of the nature of recognition memory. We highlight two distinct circuits; a mediodorsal thalamic hub, mediating familiarity, and a hippocampal–MTL cortex–ventral stream network mediating novelty, which interact via PFC and parietal neocortex. Critically, we argue that novelty is heterogeneous, with different types of novelty being defined by the kind of information that is novel in each case. Thus, we propose the distinction between absolute and contextual novelty, which, we argue, trigger different novelty detection mechanisms. Within the novelty network, the anterior hippocampus plays a critical role in detecting novelty and in triggering different types of novelty salience evaluation, depending on the type of novelty detected. We argue that contextual novelty generates dopaminergic and noradrenergic input to the hippocampus, while absolute novelty relies on cholinergic input to the hippocampus. Both of these triggers encoding mechanisms, which result in the formation of associative memories that are reported as instances of recollection at retrieval. Finally, we argue that these hippocampal neurotransmitter-mediated encoding mechanisms tune the hippocampus into an encoding-related algorithmic mode, and away from a retrieval mode.
